# Spot urine sodium as a marker of urine dilution and decongestive abilities in acute heart failure

**DOI:** 10.1038/s41598-024-51744-x

**Published:** 2024-01-17

**Authors:** Mateusz Guzik, Gracjan Iwanek, Marat Fudim, Robert Zymliński, Dominik Marciniak, Piotr Ponikowski, Jan Biegus

**Affiliations:** 1 Institute of Heart Diseases, Wroclaw Medical University, Wroclaw, Poland; 2 Division of Cardiology, Duke University Medical Center, Durham, NC USA; 3 Duke Clinical Research Institute, Durham, NC USA; 4 Department of Drugs Form Technology, Faculty of Pharmacy, Wroclaw Medical University, Wroclaw, Poland

**Keywords:** Cardiology, Medical research

## Abstract

The decongestion ability in response to diuretic treatment plays a crucial role in the treatment of acute heart failure. This effectiveness is evaluated through the assessment of sodium concentration and urine volume, which are also treatment goals themselves. However, the bidirectional interconnection between these factors remains not fully understood. The objective of this study is to provide mechanistic insights into the correlation between spot urine sodium concentrations (UNa^+^) and urine dilution. This aims to better understand of the decongestive abilities in acute heart failure (AHF). The study was single-center, prospective, conducted on a group of 50 AHF patients. Each participant received a standardized furosemide dose of 1 mg per kg of body weight. Hourly diuresis was measured in the first 6 h of the study, and urine composition was assessed at predefined timepoints. The study group presented the exponential (rather than linear) pattern of relationship between UNa^+^ and 6-h urine volume, whereas relationship between eGFR and 6-h urine volume was linear (*r* = 0.61, *p* < 0.001). The relationship between UNa^+^ and all other analyzed indices of urine dilution, including the change from baseline in urine creatinine concentration, urine osmolarity, and urine osmolarity corrected for urine sodium, also exhibited an exponential relationship. Patients who were chronically exposed to furosemide demonstrated a significantly lower urine dilution (1.78 [1.18–3.54] vs 11.58 [3.9–17.88]; *p* < 0.001) in comparison to naïve individuals. In conclusion, it should be noted that in AHF higher UNa^+^ is associated with disproportionally higher urine dilution, and patients naïve to furosemide have significantly greater ability to dilute urine when compare to chronic furosemide users.

## Introduction

Congestion stands out as the predominant cause for hospital admissions due to acute heart failure (AHF)^1^. Loop diuretics, which increase diuresis, spot urine sodium (UNa^+^) concentration and natriuresis, are the first-line drugs used to treat fluid overload^2,3^. Due to fact that spot urine sodium (UNa^+^) been shown as a reliable marker of decongestive abilities in AHF^4–6^, European guidelines and position papers recommends use this marker as an early and primary tool for assessing diuretic response^2,3^. It is intuitively and widely believed that the relationship between UNa^+^ and urine volume is linear, implying that higher UNa^+^ should correspond to a proportional increase in urine volume. Furthermore, sodium excretion is considered a therapeutic target in AHF, as sodium retention leads to passive water accumulation and congestion development^7,8^. However, the precise nature of the bidirectional link between UNa^+^ and urine volume in AHF remains incompletely elucidated. Thus, our study aims to provide mechanistic insight into the relationship between UNa^+^, urine dilution, and urine volume in the AHF population.

Our objective is to investigate the interplay between spot urine sodium, urine volume, and urine dilution in AHF patients. We believe that our findings will enhance understanding of the mechanisms underlying diuretic response and decongestive abilities in this patient population, which we hope to improve their clinical management.

## Results

### Baseline characteristics

A total of 50 patients were included in the study, of which 46 (92%) were male, had an average age of 65 ± 14 years, 22 (44%) had de novo AHF. At baseline, the mean ± SD systolic blood pressure (SBP) was 120 ± 20 mmHg, while the left ventricle ejection fraction (LVEF) was 36 ± 14%. The median [upper and lower quartile] NT-proBNP value was 6871 [4488–12564] pg/mL. The serum creatinine, estimated glomerular filtration rate (eGFR), and serum sodium were 1.48 ± 0.62 mg/dL, 58 ± 25 mL/min/1.73m2, and 140 ± 5 mmol/L, respectively. The median serum osmolarity was 295 [290–300] mOsm/L. The median urine creatinine and urine osmolarity at baseline were 77 [51–122] mg/dL and 400 [346–506] mOsm/L, respectively, the mean urine sodium was 69 ± 44 mmol/L. The mean dose of furosemide administered to the patients was 95 ± 20 mg. The general characteristics of study group was presented in Table 1.

**Table 1:** General study population characteristics.

Parameter	Value
Gender, male (*N*; (%))	46 (92%)*
Age (years)	65 ± 14**
AHF de novo (*N*; (%))	22 (44%)*
SBP (mmHg)	120 ± 20**
LVEF (%)	36 ± 14**
NT-proBNP (pg/mL)	6871 [4488–12564]***
Serum creatinine (mg/dL)	1.48 ± 0.62**
eGFR (mL/min/1.73m^2^)	58 ± 25**
Serum sodium (mmol/L)	140 ± 5**
Serum osmolarity (mOsm/L)	295 [290–300]***
Urine creatinine (mg/dL)	77 [51–122]***
Urine sodium (mmol/L)	69 ± 44**
Urine osmolarity (mOsm/L)	400 [346–506] ***
Furosemide dose (mg)	95 ± 20**

The variables were described as: * quantity (%), **mean ± standard deviation (SD), ***median [interquartile range (IQR)]. AHF—acute heart failure, SBP—systolic blood pressure, LVEF—left ventricle ejection fraction, eGFR—estimated glomerular filtration rate.

### The general diuretic response in the population

**Figure 1:**
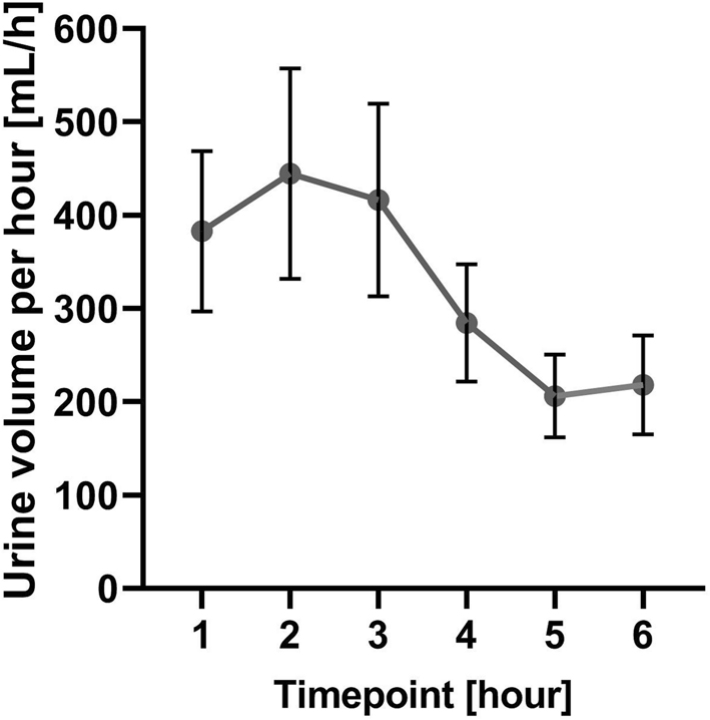
The urine volume excretion in following timepoints (mean ± 95% CI). Legend: Dot—means; vertical bar—confidence interval.

The general diuretic response of the population was illustrated in Fig. 1, which shows that the peak urine output was observed 2 h after the administration of loop diuretics. The mean urine volume during the first 6 h was 1951 ± 1352 mL, which equated to 325 ± 225 mL per hour. The mean cumulative urine volume during the 24-h collection period was 3000 ± 1585 mL.

### The relation between spot urine sodium and urine volume

**Figure 2:**
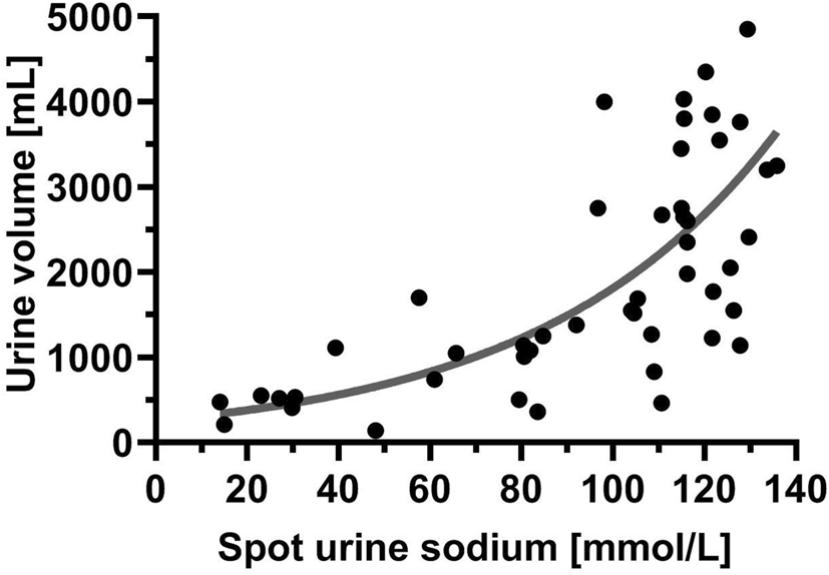
The relationship between 2nd hour spot urine sodium and 6-h urine volume. Legend: dot—absolute value; grey line—trend line.

The Fig. 2 shows the relationship between UNa^+^ levels measured at the 2nd hour of the study and cumulative urine output from 6 h after furosemide administration. This relationship appears to follow an exponential pattern rather than a linear one. From a clinical perspective, this means that lower spot urine sodium is associated with relatively lower urine output, while higher UNa^+^ is associated with much higher urine output (that is not proportionate to the increase in UNa^+^ levels). A similar pattern of relationship was observed when analyzing the relationship between UNa^+^ at all available timepoints within the first 6 h of treatment and the corresponding 6-h urine volumes (as shown in Fig. 1 in the supplement).

### The relation between baseline eGFR and urine volume

**Figure 3:**
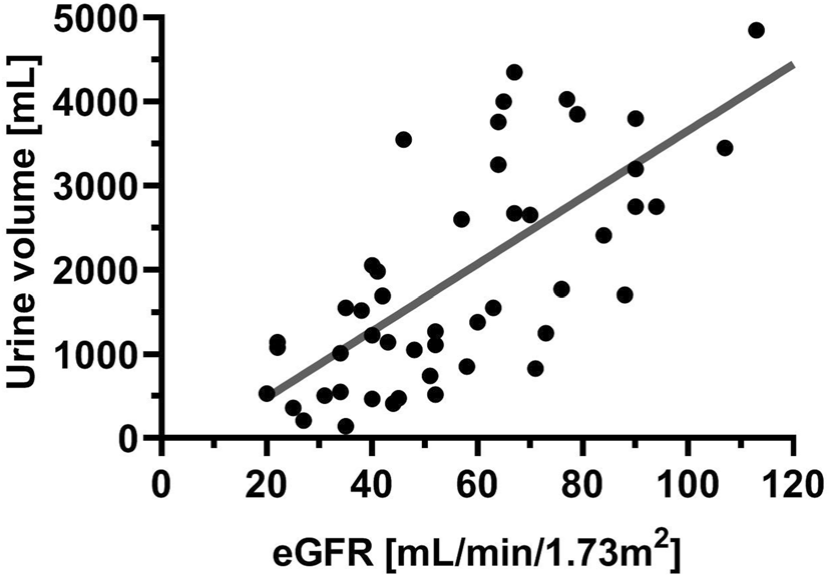
The relationship between eGFR and 6-h urine volume. eGFR—estimated glomerular filtration rate. Legend: dot—absolute value; grey line—trend line.

The relationship between eGFR at baseline and 6-h urine volume was linear (*r* = 0.61, *p* < 0.001; Pearson correlation) (Fig. 3). It means that diuretic response increased with eGFR increasing. That pattern was also observed when the naïve and chronic furosemide users were considered separately (*r* = 0.47, *p* = 0.011; *r* = 0.45, *p* = 0.036, respectively, Pearson correlation).

### The relation between spot urine sodium at 2nd hour and different markers of urine dilution

**Figure 4:**
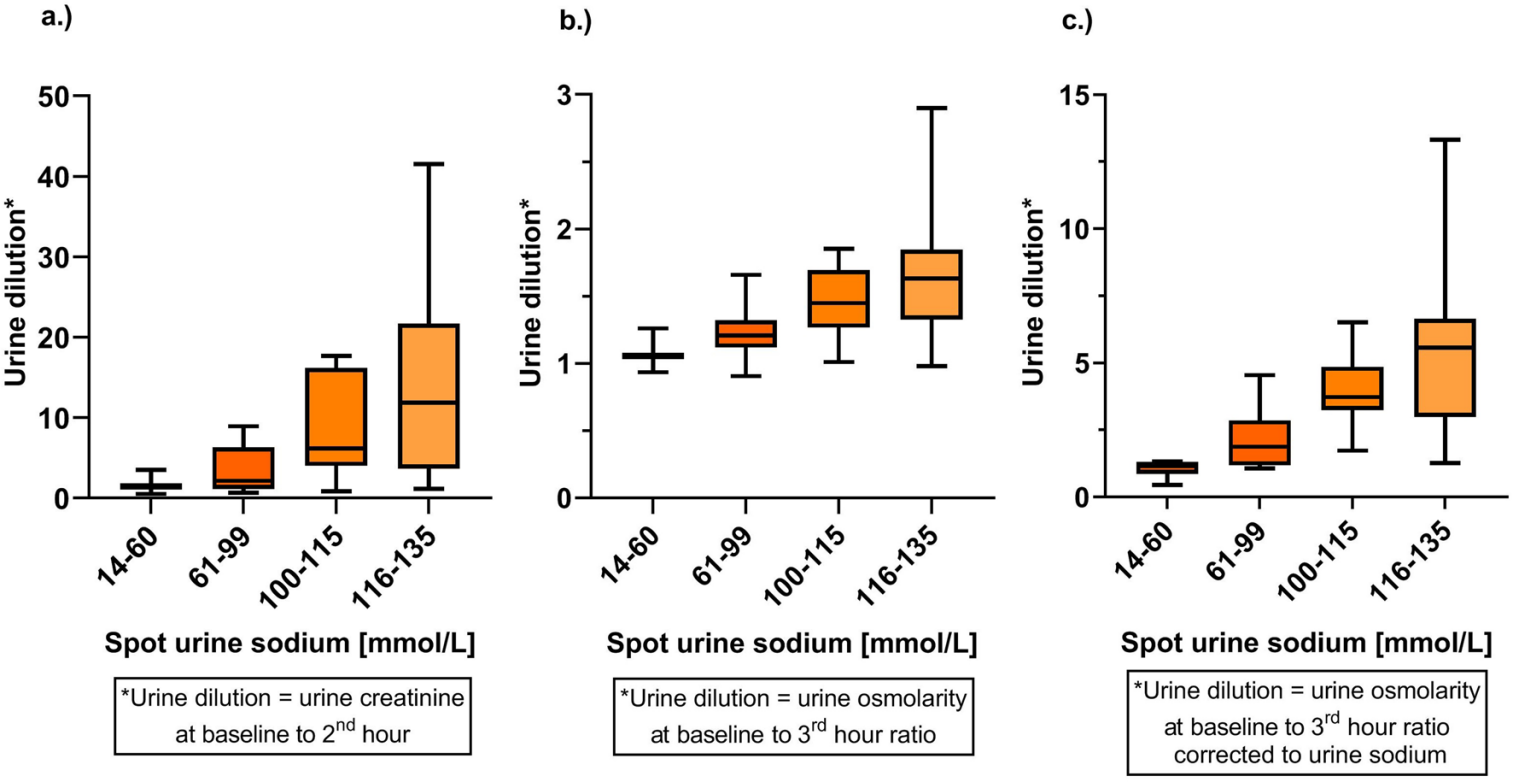
The relationship between spot urine sodium and urine dilution defined as baseline to 2nd hour urine creatinine ratio (**a**), baseline to 3rd hour urine osmolarity ratio (**b**), baseline to 3rd hour urine osmolarity ratio corrected for urine sodium (**c**) (all: median [IQR]—min to max value). Legend: horizontal line—median; box—interquartile range; vertical bar—minimal to maximal value.

Figure 4 illustrate the relationship between urine sodium concentration at the 2nd hour after loop diuretic administration (grouped in four groups: 15–60, 61–99, 100–115, and 116–136 mmol/L) and urine dilution.

The results showed that higher UNa^+^ levels were associated with greater urine dilution. The baseline urine creatinine to 2nd hour concentrations ratio increased across UNa^+^ groups (1.41 [1.18–1.87]; 2.16 [1.22–5.46]; 6.19 [4.04–16.17]; 11.89 [3.66–21.72], respectively, *p* < 0.001, ANOVA test) (Fig. 4a).

Although the serum osmolarity at baseline was similar in all UNa^+^ groups (296 [293–304]; 295 [292–297]; 300 [286–307]; 292 [290–296] mOsm/L, respectively, *p* = 0.648, ANOVA test), the baseline urine osmolarity increased across groups (328 [325–344]; 371 [361–399]; 447 [394–554]; 506 [401–561] mOsm/L, respectively, *p* < 0.001, ANOVA test). However, after three hours since the beginning of furosemide administration, the urine osmolarity became relatively equalized between these groups 312 [312–312]; 292 [291–321]; 313 [303–327]; 304 [295–317] mOsm/L, respectively, *p* = 0.322, ANOVA test), therefore the urine dilution expressed as a change of urine osmolarity (baseline to 3rd hour ratio) increased with UNa^+^ increase (1.05 [1.04–1.07]; 1.21 [1.12–1.27]; 1.45 [1.27–1.69]; 1.63 [1.33–1.85], respectively, *p* < 0.001, ANOVA test) (Fig. 4b). That relationship was even better evident when corrected urine osmolarity was applied (1.14 [0.90–1.30]; 1.88 [1.21–2.68]; 3.72 [3.25–4.85]; 5.56 [2.98–6.65], respectively, *p* < 0.001, ANOVA test) (Fig. 4c).

Abovementioned data indicate that patients with higher UNa^+^ levels at 2nd hour had a reserve to dilute the urine once exposed to loop diuretics.

### The relation between spot urine sodium and urine dilution in furosemide naïve vs chronic furosemide users

**Figure 5:**
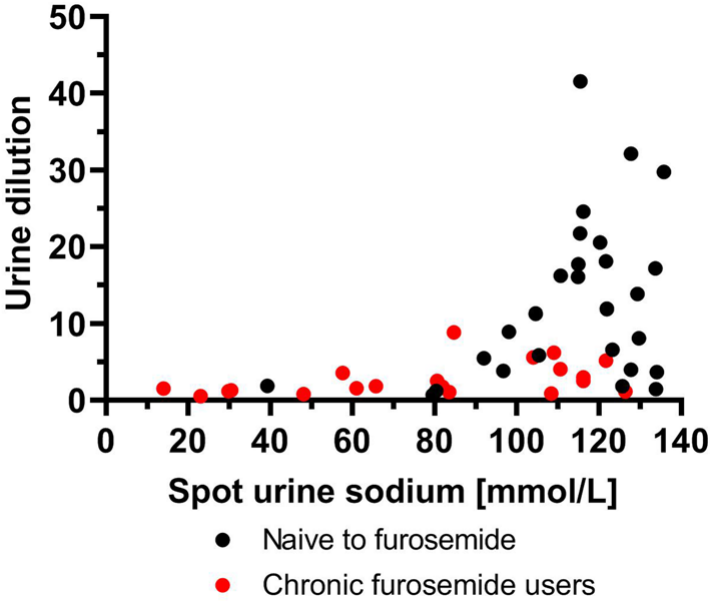
The relationship between spot urine sodium and urine dilution defined as baseline to 2nd hour urine creatinine concentration—naïve vs chronic furosemide users. Black dots—naives to furosemide, red dots—chronic furosemide users. Legend: dot—absolute value.

Chronic furosemide users had significantly lower urine dilution and 2nd hour natriuresis in comparison to naïve individuals (1.78 [1.18–3.54] vs 11.58 [3.90–18.63]; *p* < 0.001, Mann–Whitney *U*-test) and 73 ± 38 vs 113 ± 21 mmol/L; *p* < 0.001, respectively, Mann–Whitney *U*-test). It seems that despite having relatively high UNa^+^ levels, chronic furosemide users were unable to properly dilute their urine (Fig. 5).

### The relation between eGFR and urine dilution in furosemide naïve vs chronic furosemide users

**Figure 6:**
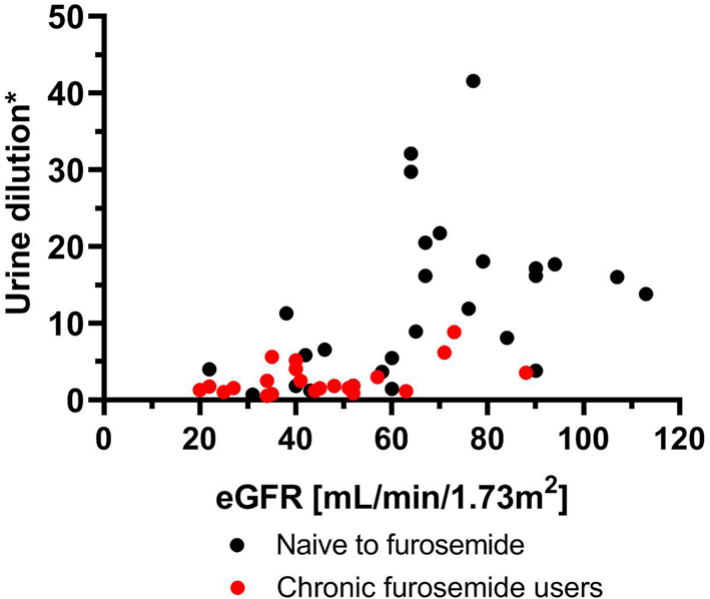
The relationship between eGFR and urine dilution defined as baseline to 2nd hour urine creatinine concentration—naïve vs chronic furosemide users. Black dots—naives to furosemide, red dots—chronic furosemide users. eGFR—estimated glomerular filtration rate. Legend: dot—absolute value.

Patients chronically on furosemide had significantly lower baseline eGFR when compared to the naïve group (45 ± 17 vs 69 ± 26 mL/min/1.73m^2^, respectively, *p* < 0.001, *t*-test) and they presented lower urine dilution (1.78 [1.18–3.54] vs 11.58 [3.9–17.88], respectively, *p* < 0.001, Mann–Whitney *U*-test) (Fig. 6).

### Modeling of relations urine sodium concentration, eGFR and 6-h urine output

**Figure 7:**
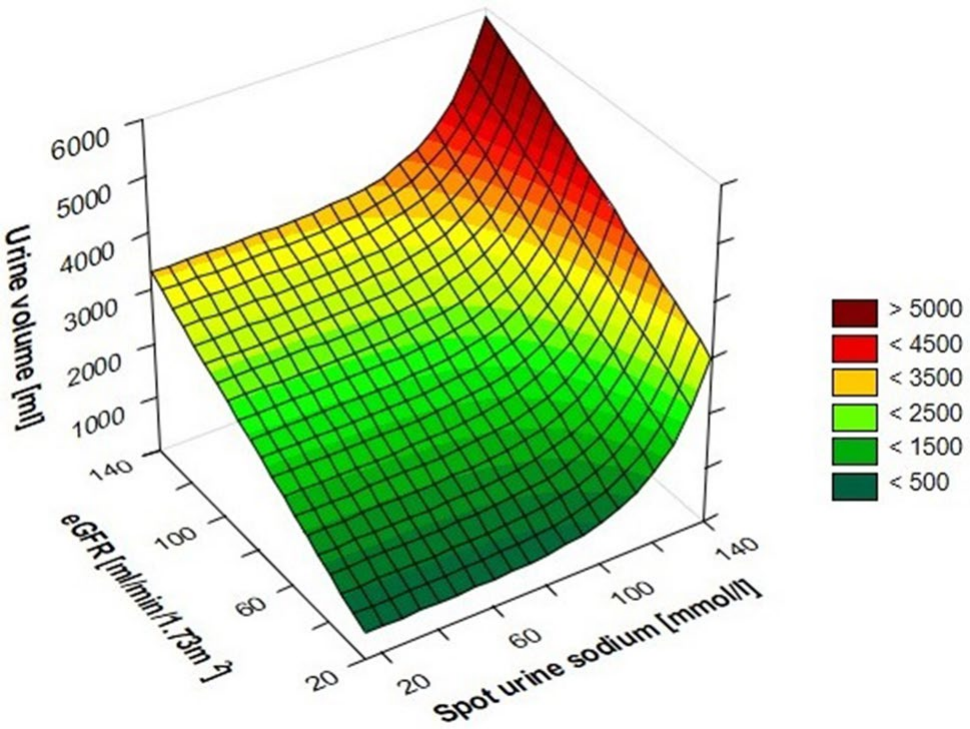
The modeling of relationship between UNa^+^, baseline eGFR and urine output at 6 h. UNa^+^—spot urine sodium, eGFR—estimated glomerular filtration rate.

The modeling of relationship between UNa^+^ at 2 h, baseline eGFR and urine output at 6 h is presented in Fig. 7.

## Discussion

The term “urine dilution” was defined through three different approaches: firstly, by considering the physiological premise that creatinine is primarily excreted via glomerular filtration, with only minimal involvement of renal tubules^9,10^. Therefore the ratio of urinary creatinine concentration before diuretic administration to its concentration at the 2nd hour of treatment was calculated. It consequently indicates that a reduction in urinary creatinine concentration signifies an increase in urine volume, reflecting the potential for an increase urine volume. This parameter allows for autonomize the kidney's response to diuretic treatment from the baseline "non-diuretic excretory function of the kidney". However, it does not account for the influence of individual osmotically active substances on urine dilution, which may deviate from physiological norms in AHF and depends on the function of the kidneys' tubular channels in urine formation. This measure specifically addresses the overall kidney's ability to increase the volume of excreted urine in response to loop diuretic treatment. In light of this, urine osmolarity change was performed as a second and (after urine sodium correction) third method. This approach was employed to elucidate alterations in urine volume concerning the excretion of osmotically active agents compared to the “pre-diuretic” state—the second method encompassing all osmolytes and the third method focusing on osmolytes other than spot urine sodium. This approach emphasizes the mechanistic aspect of the potential to diuretic response and seems to be familiar with the clinical aspect of this issue.

The most important finding of our study pertains to the description of diuretic response patterns and their relation with urine sodium concentration in the AHF population. Within the entire cohort of AHF patients, it became evident that the relationship between spot urine sodium levels and urine output adheres to an exponential pattern rather than a linear one, challenging intuitive assumptions. From a clinical perspective, this implies that lower urine sodium levels are associated with relatively lower urine output due to lower urine dilution, whereas higher urine sodium levels are associated with significantly higher urine output, which is not proportionate to the increase in spot urine sodium and results in greater urine dilution.

Upon conducting a thorough analysis of diuretic response across the entire study population, a notable disparity emerged in the ability to respond to loop diuretics between patients chronically on furosemide and those furosemide-naïve. Patients chronically exposed to furosemide presented a blunted response across UNa^+^ increasing, contributing to the lower part of the dilution-UNa^+^ curve, while the furosemide-naïve group demonstrated a robust response, responsible for the steep incline of the curve. Therefore, in general population of AHF patients the relationship between urine output and UNa^+^ presents an exponential shape, which is key to understanding the mechanism of decongestion (process of relieving symptoms and signs of volume overload in vascular or tissue compartment in AHF).

It is crucial to emphasize that we do not dispute the role of urine sodium in urine formation (and its early predictive role in urine output). Instead, we try to extend our understanding of the interplay between urine biomarkers and urine volume, dilution, which may have clinical applications.

We demonstrate that chronic exposure to furosemide not only relates to known nephron anatomical adaptations but also leads to a diminished ability to dilute urine^8,11,12^. As with given urine sodium concentration the patients (naïve vs chronic furosemide users) had different urine dilution and urine volumes, elucidating why patients with a low UNa^+^ response to loop diuretics (at 2nd hour) have limited potential for decongestion.

Additionally, we observed that despite an increasing baseline urine concentration (expressed as urine osmolarity) across UNa^+^ groups before furosemide administration, the urine osmolarities became equal in the 3rd hour of the study between the groups. This indicates that the drop in urine osmolarity was greater in the subpopulation with high UNa^+^. Hence, the large group of patients with UNa^+^ levels over 80–100 mmol/L exhibits the ability for rigorous urine dilution. However, within that group, the dispersion of the dilution indexes was the highest, suggesting a potential issue in patients with relatively high UNa^+^ levels but low urine dilution.

The challenge of patients with satisfactory natriuresis but poor urine dilution may be partially explained by the fact that individuals with this profile have predominantly been chronically exposed to furosemide. During chronic loop diuretic use, compensatory mechanisms develop in different parts of the nephron, including the upregulation of different sodium channels responsible for these alterations^13^.

Interestingly, the relationship between eGFR and renal response (in terms of urine volume and dilution) was linear in the entire group. This pattern did not align with the observed relationship between natriuretic response and urine output, providing further evidence for at least a partial dissociation between eGFR and natriuretic response in AHF, as previously postulated^5^.

Recent studies have shown promising results for SGLT-2 inhibitors in treating AHF patients^14–17^. This drug class may possess decongestive properties, potentially leading to urine dilution through osmotic diuresis. Consequently, based on the example of SGLT-2 inhibitors, it can be hypothesized that alternative, sodium-independent pathways of urine dilution may be explored and utilized for decongestion in the future. This suggests that individuals with disrupted sodium-dependent urine dilution mechanisms may benefit from the action of hypothetical sodium-independent ones. However, it is essential to remember that the use of certain active particles, such as vasopressin antagonists causing water excretion, did not result in improvements in HF outcomes^18^.

Our study was also subject to certain limitations. It was single-center, non-randomized trial which could have limitations in terms of generalizability to broader populations and potential for bias due to the lack of randomization. In addition, using a standardized furosemide dose allows for better comparisons between different patients, but on the other hand, it may not accurately reflect the individual needs of each participant. The limited number of patients is another obvious limitation. We are also aware that there are several confounding factors that may impact the final diuretic response, potentially introducing bias. The lack of the strict diet protocol may also interfere with the presented results. Therefore, the findings should be interpreted with caution and further research is needed to validate these results. The assessment of presented results may be conducted in randomized study, on larger and more diverse population. Also more individualized approach to diuretic dosing, with unifying diet during study and fluid intake may be valuable.

In conclusion, it should be noted that in AHF, higher UNa^+^ is associated with disproportionally urine dilution. Patients naïve to furosemide have significantly greater ability to dilute urine when compared to chronic furosemide users.

## Methods

This was a single-center, prospective, observational study conducted between June 2021 and April 2022 at the Institute of Heart Diseases, Wroclaw Medical University, Poland. AHF was defined according to the European Society of Cardiology (ESC) guidelines criteria^2^. The study was approved by the Wroclaw Medical University Ethical Comitee (number of consent: 261/2021) and conducted in accordance with the Declaration of Helsinki.

## Study population

All adult patients hospitalized with AHF who were willing to participate were screened for the study. Patients who met the inclusion criteria, did not meet any of the exclusion criteria, were enrolled to the study. All participants provided the written informed consent form to the study.

The inclusion criteria were as follows:

- Primary diagnosis of AHF with NTproBNP > 2000 pg/mL and clinically overt fluid overload (lower extremity edema reaching at least knees) requiring intravenous furosemide
- Study enrollment within 36 h of hospital admission to ensure all patients were in the active decongestive phase of AHF, as urine Na^ +^ and diuretic response may vary during different phases of AHF^19,20^.
- Washout period of ≥ 6 h from the last dose of intravenous (or oral) furosemide if the patient received loop diuretic before the baseline procedures of the study

The exclusion criteria were as follows:

- Active infection (C-reactive protein > 50 mg/L, procalcitonin > 0.1 ng/mL) or receiving antibiotics
- Acute coronary syndrome
- End-stage renal disease requiring renal replacement therapy
- Cardiogenic shock
- Recipient of contrast media within the last 72 h

Data collection included demographics, clinical history, co-morbidities, and previous therapies. Physical findings were also recorded. As part of routine clinical practice at our institution, all patients were instructed to limit their fluid intake to 1.5–2 L per 24 h and advised to limit their daily sodium intake during hospitalization. Patients were also asked to eat only the food provided by the hospital, however the additional meals were not forbidden.

### Laboratory assessment of blood and urine

During the study, several laboratory assessments were performed using standard methods. Additionally, the following laboratory tests were performed:

- Plasma NT-proBNP (N-Terminal Pro-B-Type Natriuretic Peptide) was measured at baseline and 24 h using an immunoenzymatic method (Siemens kit from Marburg, Germany).
- eGFR (estimated glomerular filtration rate) was calculated by MDRD equation
- Urine composition (including Na^+^, K^+^, Cl^−^, creatinine, and urea) was measured at baseline, 1st h, 2nd h, 3rd h, 6th h, and 24th h.
- The urine osmolarity was measured at baseline and at 3rd h of study.

To perform the analysis, the study population was divided based on their UNa^+^ measure at 2nd h into four groups: 15–60 mmol/L, 61–99 mmol/L, 100–115 mmol/L, and 116–135 mmol/L. Additionally, the population was stratified based on their exposure to chronic furosemide, into naïve users and chronic furosemide users.

“Urine dilution” was the term defined by authors to evaluate kidney potential to increase urine excretion in response to loop diuretic treatment. It was assessed using three separate methods:

- Based on urine creatinine, by calculating the proportion of urine creatinine concentration at baseline to the 2nd h values,
- Based on urine osmolarity by assessing the urine osmolarity change ratio between the baseline and 3rd h.
- Based on corrected urine osmolarity for urine sodium by assessing the urine osmolarity change ratio between the baseline and 3rd h timepoints (to calculate the corrected osmolarities we have mathematically removed the sodium contribution from the equation).

### The study procedures

All eligible patients underwent baseline procedures that commenced between 7:30 and 8:30 AM (including blood samples, and weight measurement) and then received a standardized dose of furosemide (see below). Morning oral medications were administered before furosemide administration, and no new drugs were permitted during the first 6 h of the study to minimize confounding factors. Blood samples were collected at baseline, 3 h, 6 h, and 24 h, while exact urine output was recorded hourly during first 6 h, then 24-h collection was continued. Urine composition assessments were conducted at baseline, 1st h, 2nd h, 3rd h, and 6th h, with samples obtained from the cumulative urine collection in the previous hour. The urine collection was continued from 6th to 24th h, during which all medications and intravenous infusions were permitted if necessary. Only patients who fulfilled the criteria for "rescue furosemide" were allowed to receive additional furosemide during this phase (see below). The baseline, early morning urine samples obtained after at least a 6-h washout period were used as a reference for comparison of urine dilution after furosemide administration. To ensure comparable doses of furosemide, patients received protocol-driven (standardized) diuretic therapy, with the dose calculated as 1 mg per 1 kg of body weight. The half of the total dose of furosemide was administered as a bolus to achieve a high drug's serum concentration, followed by the other half dose administered as a 2-h infusion. Patients with a poor diuretic response during the first phase of the study, defined as urine output < 100 mL/h at the 5th and 6th h, were allowed to receive an additional 40 mg of furosemide after the first 6-h period of the study.

## Statistical analysis

Continuous variables with a normal distribution were presented as mean ± standard deviation (SD), while skewed variables were presented as median with [interquartile range], categorical variables were reported as number and (percentages). The normality of distributions and equality of variances were checked using the Shapiro–Wilk test and Levene's test, respectively. Differences between groups were evaluated using the *t*-test, Mann–Whitney *U*-test, ANOVA test, or Kruskal–Wallis test (with post hoc tests when appropriate). Welch correction was used when compared groups with normal distribution differed in variances. Correlations between variables were assessed using Pearson correlation tests, as well as multivariable regression analyses. To perform the model of urine volume dependence on UNa^+^ and eGFR the non-linear regression model was used with formula:

$${\text{Urine volume}} = {\text{A*}}e^{{B{*}\left( {UNa^{ + } } \right)}} + C{*}eGFR + D$$

where:

A, B, C, D—constants calculated based on empirically obtained data

$${\text{UNa}}^{ + } {-\!\!-}{\text{urinary sodium concentration}}$$

$${\text{eGFR}}{-\!\!-}{\text{estimated glomerular filtration rate}}$$

*P*-value of < 0.05 was considered statistically significant. Statistical analyses were performed using STATISTICA 13 (StatSoft), and graphical representation was created using GraphPad Prism.

### Supplementary Information

Supplementary Figure 1.

## Data Availability

The datasets used in the current research are available from the corresponding authors on reasonable request.
