# Author Correction: Spot urine sodium as a marker of urine dilution and decongestive abilities in acute heart failure

**DOI:** 10.1038/s41598-024-59807-9

**Published:** 2024-04-18

**Authors:** Mateusz Guzik, Gracjan Iwanek, Marat Fudim, Robert Zymliński, Dominik Marciniak, Piotr Ponikowski, Jan Biegus

**Affiliations:** 1 Institute of Heart Diseases, Wroclaw Medical University, Wroclaw, Poland; 2 Division of Cardiology, Duke University Medical Center, Durham, NC USA; 3 Duke Clinical Research Institute, Durham, NC USA; 4 Department of Drugs Form Technology, Faculty of Pharmacy, Wroclaw Medical University, Wroclaw, Poland

Correction to: *Scientific Reports* 10.1038/s41598-024-51744-x, published online 17 January 2024

The original version of this Article contained an error in the Acknowledgements section. The subsidy number was incorrect.

“This research was financially supported from the subsidy no. No. AZP-261-W-230/23 for the Institute of Heart Diseases, Wroclaw Medical University, Poland.”

now reads:

“This research was financially supported from the subsidy no. SUBZ.A460. 23.005 for the Institute of Heart Diseases, Wroclaw Medical University, Poland."

The original Article has been corrected.

