# Supplementary material for: Spot urine sodium as a marker of urine dilution and decongestive abilities in acute heart failure
Source: Sci Rep. 2024 Jan 17;14:1494. doi: 10.1038/s41598-024-51744-x (PMC10794205; doi:10.1038/s41598-024-51744-x)
Supplement: Supplementary file 1 — Supplementary Figure 1. [file 41598_2024_51744_MOESM1_ESM.docx]

Spot urine sodium as a marker of free water clearance and decongestive abilities in acute heart failure.

*Understanding spot urine sodium in acute heart failure.*

SUPPLEMENTARY MATERIAL


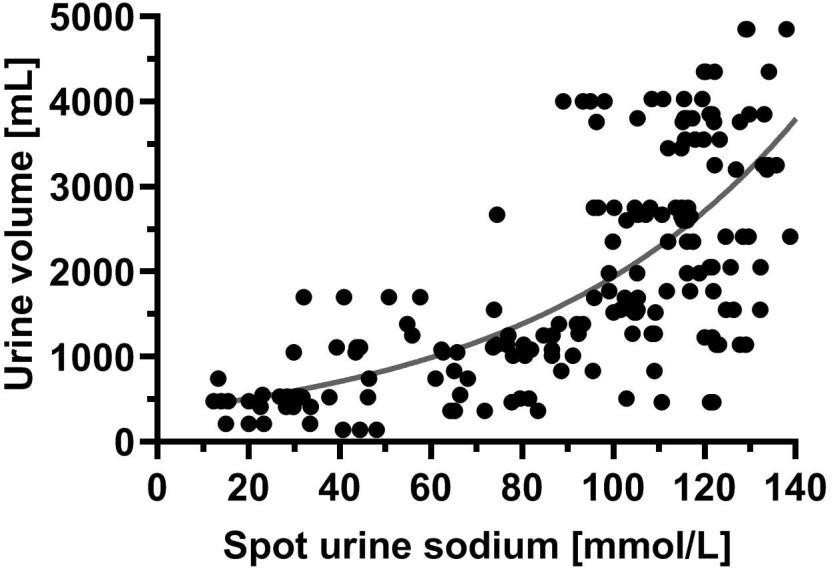


**Supplementary material - figure 1.** The relationship of summarized all timepoints spot urine sodium and 6 hours urine volume. *Legend: dot – absolute value, grey line – trend line*
